# The Guatemalan Construction Industry: Approach of Knowledge Regarding Work Risks Prevention

**DOI:** 10.3390/ijerph15102252

**Published:** 2018-10-15

**Authors:** Francisco Arturo Hernández-Arriaza, José Pérez-Alonso, Marta Gómez-Galán, Ferdinando Salata

**Affiliations:** 1 Escuela Mecánica Industrial, Faculty of Engineering, University of San Carlos, Edificio T-1, Ciudad Universitaria Zona 12, Guatemala C.A. 01012, Guatemala; faha04@gmail.com; 2 Department of Engineering, University of Almería, Agrifood Campus of International Excellence (CeiA3), 04120 Almería, Spain; mgg492@ual.es; 3 Department of Astronautics, Electrical and Energetics Engineering, University of Rome “Sapienza”, 00184 Rome, Italy; ferdinando.salata@uniroma1.it

**Keywords:** Guatemala, health and safety in the workplace, prevention, contractor, construction companies, multiple correspondence analysis

## Abstract

In the present work, the results are presented for the characterization of work risk prevention in the Guatemalan construction industry. This characterization has been carried out using a simple random sampling technique, employing a questionnaire that was structured into 3 groups of variables: 1. General company data; 2. Prevention and management activities regarding health and safety in the company and on the worksite; and 3. Health and safety in the contractor companies. Following the sampling phase, the data were introduced in a database format, and a preliminary analysis was performed on the studied variables, followed by a descriptive analysis and a multiple correspondence analysis. The main findings of the study emphasize that companies in the Guatemalan construction sector are characterized as dedicating most of their activity (52.0%) indistinctly between civil engineering work, building construction and other specialized construction, mainly working as contractors (47.5%). These are “medium-sized” companies, employing an average of 81.1 on-site workers, having an average of 6.8 on-site work crews, and grossing an average turnover of 1.29 million euros annually. Likewise, it found that the larger construction companies adopt better prevention and management measures for worksite health and safety the larger companies are correlated with a high awareness of experiencing worksite accidents, while medium-sized companies have medium-level awareness. Companies with fewer workers manage workplace risk prevention worse, with low accident risk awareness. This correlation between these indicative variables of company size and workplace risk management and prevention is clearly reflected in the four company “clusters” that have been identified as having homogenous characteristics using the multiple correspondence analysis technique. Companies in the Guatemalan construction sector should make a greater effort to improve manager and worker training regarding workplace risk prevention to increase the effectiveness of company prevention management.

## 1. Introduction

The construction sector registers very high labor accident rates in relation to the indices of other activity sectors [1,2,3,4,5,6,7] and as a consequence, there are high costs associated with work accidents that may depend on the culture, the risk level, the number of operators and the participation of subcontractors on each project [8,9,10,11]. Even López-Valcarcel [12] has written that more than half of all workplace injuries and deaths worldwide are attributed to, or are the responsibility of, the construction industry [13].

The culture of many workers helps to explain the high incidence rates in the construction industry. Factors such as machismo, substance abuse, language barriers and the low level of education are some of the most relevant aspects related to the worker’s culture [14]. According to Hinze and Wiegand [15], the attitude of construction workers increases risk tolerance and, therefore, the frequency and severity of accidents. Two main reasons have been used to explain this high accident rate in the construction industry [14]: (1) the intrinsic risk due to the nature of the activities and the particular characteristics of the construction projects and organizations and (2) the financial and economic issues related to the implementation of additional security measures in a competitive growing market.

Therefore, the internal information on the cost of accidents generated during the work’s execution, as well as the phases in which they are produced, could help in making decisions regarding safety [8]. In this sense, Arévalo indicates that knowing the economic repercussion of accidents, from the design phase and its prevention measures, would make it possible to delimit a more efficient security management system in the company from its origin, as well as deepen the prevention and study of accidents, and provide enough personal protection equipment (PPE) [16]. Complying with PPE use and availability, which may depend on the size of the organization and the type of training for its use [17], as well as other security measures, is required to reduce injuries and fatal accidents among construction workers [18,19,20] or among visitors to the construction site [21,22].

Safety is a process that considers the use of all types of tools and techniques to systematically guarantee worker’s well-being [23,24,25]. Thus, the main factors that affect safety in construction companies are poor safety awareness by top management, poor training, poor safety awareness by safety coordinators and drafters of projects, reluctance to provide safety resources and reckless operations [26].

To prevent accidents, exhaustive maintenance is necessary along with periodic safety inspections, continuous safety training and the development of an accident investigation plan [27]. Therefore, there are a wide variety of works that investigate the causes of construction accidents and affect the safety of workers, such as the company size, its safety policy, the attitude of workers, preventative coordination at the project and execution phase of the planned work [15,28,29], and safety management in the company [26,28,30]. Thus, for example, the causes of accidents that occurred in construction and their relationship with the construction phase in which they occur are analyzed [29].

Prevention ought to be considered from the moment a professional initiates a workplan or starts a job, involving designers and contractors [31,32,33], giving the importance of the safety and health coordinator’s role, for their participation in the distinct phases of the project [5]. Therefore, it is recommended that the safety and health management in construction projects present an integrated approach, so that it involves all stages of the project’s life cycle: design, execution, and operation. In this sense, a lot of researches have been carried out on the risks prevention through design (Construction Hazard Prevention through Design (CHPtD)), that is, integrating worker safety prevention measures into the design phase of the project by the designers of the construction sector [34,35,36]. Another step in the prevention of risks through design is the new Building Information Modeling (BIM) system that aims not only to improve safety but also productivity [37,38,39].

As we have seen, safety and health at work in the construction industry has been and continues to be a subject of intense research and practical development. Worldwide, there has been a substantial improvement in occupational safety and health in the construction industry, generally motivated by the publication and continuous implementation of the most relevant standards in the field ILO-OSH 2001, OHSAS 18001, and ISO 45001 [40,41,42], and increasingly strict regulations [14].

However, developing countries do not adapt quickly enough to the implementation of this regulatory body, which is why they present more problems of accidents. Thus, it was not until 1957 that Guatemala established the General Law for Health and Safety in the workplace [43], and it was not until 7 October 1991 that agreement 167-1988 concerning health and safety in the building sector was approved [44]. In Guatemala, according to the Guatemalan Institute of Social Security hospital register of accidents out of the manufacturing-construction-service industries in 2016, 28.15% corresponded to construction (24.75% in 2017) [45]. As in any developing nation, the risk of having one’s health impacted is 10 to 20 times greater than in industrialized nations [13,46].

For all of the aforementioned reasons, and given that no scientific documentation exists regarding workplace risk prevention in the Guatemalan construction industry, the objective of this study is to characterize workplace risk prevention in Guatemalan construction companies in a way that correlates company size with prevention activity parameters and workplace risk management, as well as other corresponding structural and organizational parameters.

## 2. Materials and Methods

### 2.1. Research Design

**Figure 1:**
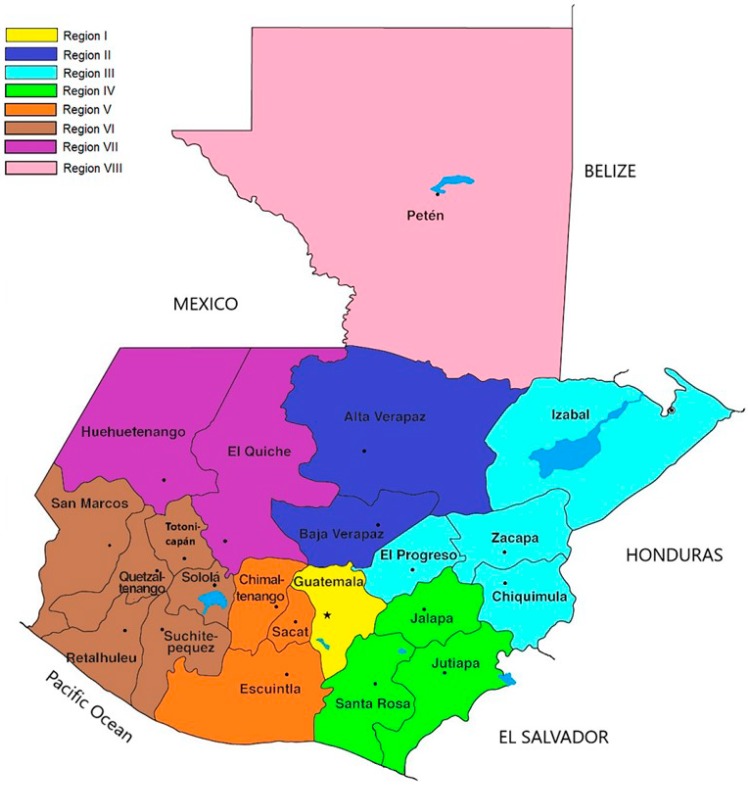
Map of the territorial division of Guatemalan departments.

To characterize workplace risk prevention in Guatemalan construction companies (see Figure 1), the quantitative and qualitative variables expounded in Table A1, Table A2, Table A3, Table A4, Table A5 and Table A6 (see Appendix A) have been adopted as study variables; these are grouped into 3 groups of homogenous variables. Only those in the first group, namely those describing company generalities, are there 5 quantitative variables (Annual company turnover (C), the number of workers in the office (D), the number of workers on site (E), the number of work crews over the year (F), and the number of years experience working on site (G)). All the other variables are qualitative. However, the quantitative variables are categorized to better study and correlate them. In Table A1, Table A2, Table A3, Table A4, Table A5 and Table A6, one can see all the variables and their nomenclature, and for the qualitative and quantitative variables categorized, their categories and nomenclature are shown.

To acquire the field data for the studied variables, a sampling was carried out of Guatemalan construction companies using a questionnaire designed for the task. Once the data were obtained, a preliminary analysis was carried out to identify absent and missing data, and then we checked that the data verified the conditions of independence, homoscedasticity, and normality of the variables. With the aim of better understanding the interrelationship between the variables, a bivariate correlation analysis was performed on the variables, calculating the Spearman correlation coefficients. Likewise, a descriptive analysis was performed on the studied variables—for the qualitative variables, the frequency for each category of each expressed variable was described in percentages, whereas for the quantitative variables, their average values were determined along with the standard deviation. Finally, multiple correspondence analysis was performed that allowed one to determine which variables, and which categories for each variable, corresponded with each other, to know if significant correspondences existed between categories of variables, and groups of companies with common characteristics.

### 2.2. Questionnaire Development

To collect the information, a specific questionnaire was designed, based on previous Spanish research works [47,48], which were structured into three sections that grouped homogenous variables. These three groups of variables were:

1. General Company Data: containing 8 variables (Table A1, see Appendix A).
2. Prevention Activities and Health and Safety Management in the Company and on the worksite; containing 38 variables (Table A2, Table A3, Table A4 and Table A5, see Appendix A).
3. Health and Safety in the Contractor Companies: containing 11 variables (Table A6, see Appendix A).

### 2.3. Sample

A simple randomized system was used to perform the sampling, considering the population to be the totality of construction companies in Guatemala that, according to the country’s National Statistics Institute was 7720 companies as of December 2015 [49]. However, to gather reference data on them, we turned to the Ministry of Communications, Infrastructure and Housing (Ministerio de Comunicaciones, Infraestructura y Vivienda (MICIVI)) which provided the database of existing prequalified companies (namely, the address, legal representative, telephone, e-mail, financial standing, and date of incorporation), which as of December 2015 totaled 1954. For the sample size extraction, with a confidence level of 95% (Z = 1.96), an acceptable error limit of 7% and a standard deviation of 5% resulted in a sample size of 178 companies. This amount is equivalent to 9.11% of registered companies. To guarantee the present research, we initially sent 5 surveys that, on being received, generated a receipt validation with small modifications in the questioning. In total, 350 surveys were sent out to Guatemalan constructors via the Internet using the Google Forms application as well as by printed paper (17.91%); this resulted in 86 being sent back to us digitally, of which 18 were removed because no more than 30% of the total survey was completed. Of the printed versions, a total of 34 were returned, of which 2 were discarded for the same reason (so as not to skew the results of the present research)—this left 100 effective samples with significant responses. The sampling period was from March 2016 to December 2017.

## 3. Results

### 3.1. Response Rate and Consistency

In accordance with the sampling plan indicated in Section 2.3, 350 surveys were sent out, of which 100 were valid; thus, the effective response rate was 28.57%, less than the 38.0% obtained by Chen and Mohamed in a sampling study of building contractors in Hong Kong [50].

To study the consistency of the variables analyzed in the present study, a correlation analysis was performed on the variables using the Spearman rank correlation coefficient for each pair of variables, because, in accordance with Pérez-Alonso and collaborators [48], this coefficient shows the level of superposition of variable fields, such that if the value is greater (e.g., greater than 0.80), multicollinearity would exist between them, and thus indicate that identical fields are represented, and that some of the statistical analysis variables should be discarded. As a result, the variables presenting correlation coefficients greater than 0.80 were discarded from the multiple correspondence analysis (MCA), thus controlling the multicollinearity of the variables, leaving the correlation coefficients for the remaining variables between 0.15 and 0.728.

### 3.2. Descriptive Analysis

In Table A1, Table A2, Table A3, Table A4, Table A5 and Table A6 (see Appendix A), the frequency for each of the qualitatively studied variable categories is shown along with those variables that were quantitatively categorized.

Regarding the quantitative variables, the companies in the sample presented an average annual turnover (C) of 1.29 million euros (s.d. 6.29), an average number of office workers (D) of 7.3 (s.d. 15.06), an average number of on-site workers (E) of 81.1 (s.d. 75.22), an average number of on-site work crews (F) of 6.8 (s.d. 5.55), and an average of 2.7 years of experience working on site (s.d. 0.55).

### 3.3. MCA

The results of the MCA performed for the representative variables (Table A7, see Appendix A) allow one to identify the correlation of the variable categories, as well as their own variables using a two-dimension model in which information regarding all analyzed variables is summarized.

#### 3.3.1. Reliability of the Multiple Correspondence Model

The model obtained after the above analysis presented two significant dimensions, in which the first explained 39.4% of the variance with a Cronbach α coefficient of 0.972 and an eigenvalue of 22.079; while the second dimension explained 26.3% of the variance with a Cronbach α coefficient of 0.949 and an eigenvalue of 14.747—so that for the joint factorial model, the average variance explained is 32.9%, the average Cronbach α coefficient is 0.963 and the average eigenvalue is 18.413, signifying that there is good model reliability.

#### 3.3.2. Discrimination Measures

In Table A7 (see Appendix A), the discrimination measures are shown for each variable regarding each of the two dimensions and the average. As can be appreciated, the lead variable in the ranking of explicative variables is AAA (0.628), since it presents the largest discrimination, followed in order of descending explanation by the variables Z (0.528), Y (0.527) and V (0.523). The least explicative variable is OO (0.024), followed by EEE (0.030), S (0.117), Q (0.118) and A (0.149). In terms of the discrimination in both dimensions, the first dimension presents very large discriminations with the variables AAA (0.805), KK (0.728), II (0.721), V (0.717), ZZ (0.695), Z (0.673), U (0.665), X (0.658), LL (0.623), M (0.621), Y (0.620) and L (0.610), while the second dimension presents large discriminations but less than those of dimension 1, with the variables E (0.639), F (0.596), TT (0.579) and C (0.487).

Each measure of discrimination coincides with the coordinate variance for each dimension of the modalities for each variable; such that the variables whose modalities have different coordinates for a dimension present elevated discrimination measures for that dimension. Likewise, discrimination measures similar to a variable in the two dimensions reflect difficulties in assigning them to a given dimension. The ideal is for a variable to have a high value in only one dimension and be low in the other, as occurs with the variables II, L, KK, LL and U, which are more correlated with dimension 1; therefore, the dimension better discriminates the categories of these variables; while the variables E, TT and F are more correlated with dimension 2—consequently, this dimension better discriminates the categories of these variables.

#### 3.3.3. Quantifications

**Figure 2:**
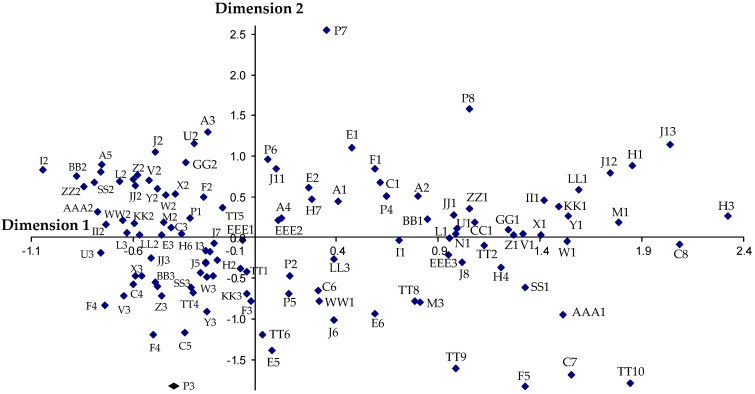
Factorial plane of the quantifications of the variable categories.

The MCA carried out allows one to identify the categories for each variable that discriminate the objects (companies) more; and thus, the quantifications of the variables are obtained and represented on a factorial plane, in which the axes are the two model dimensions (Figure 2). The quantification of the categories are the average scores for the objects of the same category; and through the representation of the factorial plane (Figure 2), we can observe the correlations and correspondences of the variable categories.

#### 3.3.4. Contribution of the Dimension to the Inertia of the Point for Each Variable

In addition to the quantifications, to discover which category for each variable best contributes to each dimension, the correspondence analysis calculates the contributions of the dimension to the inertia of the point for each of the variables, which are shown below for five general company variables and the most significant of the other variables, expressed in percentages.

For variable A, the category that best explains the positive value of dimensions 1 and 2 is to present a building construction activity exclusively (A2) (8.5% and 3.4%, respectively) and for negative values to work simultaneously in civil engineering, building construction and specialized constructions (A7) (6.3% and 9.7%, respectively), since these present the largest dimension contributions to the inertia of the point for the variable.

For variable C, the category that best expresses the positive values of dimension 1 is that the company turns over between 2–10 million euros (C7) (10%), and for negative values that it turns over between 0.1–0.3 million euros (C2) (8.1%); while for positive values of dimension 2, that it also turns over between 0.1–0.3 million euros (C2) (11.7%) and for negative values, that it turns over between 0.7–1 million euros (C5) (14.1%). Regarding variable E, the category that best accounts for the positive value of dimension 1 are companies of between 11 and 50 site workers (E2) (3.8%), and for negative values, companies of between 101 and 150 site workers (E4) (7.9%); while for positive values of dimension 2, they are companies with between 11 and 50 site workers (E2) (20.4%) and for negative values, companies with between 151 and 200 site workers (E5) (23.3%).

For the F variable, the category that best accounts for positive values in dimension 1 is that the number of work crews is less than 4 (F1) (8.5%), and for negative values, that the number of work crews is between 4 and 6 (F2) (2.0%); while for positive values of dimension 2, that the number of work crews is less than 4 (F1) (17.3%), and for negative values, that the number of work crews is between 7 and 10 (F3) (16.5%). For the H variable, the category that best explains the positive value of dimension 1 is that the company works on the job as the developer and as the contractor (H4) (10.1%), and for negative values, that it does so as the contractor and subcontractor (H6) (4.1%); while for positive values of dimension 2, that the company works on the job as the developer, contractor and subcontractor (H7) (3.8%), and for negative values, that it does so as the developer and contractor (H4) (1.0%).

With regard to variable I, the category that best expresses for positive values in dimension 1 is that the company implements a prevention plan before starting the job (I1) (32.8%), and for negative values that the company does not implement such a plan (I2) (18.6%); while for positive values of dimension 2, that the company does not implement a prevention plan (I2) (12.0%), and for negative values, that the company sometimes implements such a plan (I3) (1.8%). For variable J, the category that best accounts for positive values in dimension 1 is that the company adopts personal and collective protection, color codes and signaling (J8) (15.5%), and for negative values that they only adopt personal protection (J2) (5.2%); for positive values in dimension 2, that they only adopt personal protection as a preventative work measure (J2) (24.1%), while for negative values, that they adopt personal and collective protection and signaling (J5) (3.8%) as preventative work measures.

Regarding variable L, the category that best explains the positive values in dimension 1 is that the company gives training to new company personnel (L1) (58.5%), and for negative values, that the company sometimes gives training to new company personnel (L3) (42.6%); while for positive values for dimension 2, that the company does not give training to new company personnel (L2) (1.8%); whereas for negative values, there is no category that discriminates well. For variable M, the category that best expresses the positive values in dimension 1 is that the company performs a medical and aptitude test on new company workers (M1) (37.6%), and for negative values, that the company does not perform a medical and aptitude test on new workers (M2) (45.0%); while for positive values for dimension 2, that the company likewise does not perform a medical and aptitude test on new company workers (M2) (7.7%), and for negative values, that the company sometimes performs a medical and aptitude test on new company workers (M3) (10.6%).

For variable P, the category that best accounts for positive values in dimension 1 is that the company performs a risk and safety evaluation during the work’s execution (P4) (6.8%), and for negative values, that it carries out a risk and safety evaluation before starting the job (P1) (7.2%); while for positive values of dimension 2, that the company does not respond to the question asking when it performed a risk and safety evaluation for the job (P7) (5.3%), and for negative values, that it carried out a risk and safety evaluation before, during and at the end of the job (P5) (7.1%). For variable U, the category that best explains the positive values for dimension 1 is that the company installs toilets and hand-washing facilities on the job (U1) (59.5%), and for negative values, that the company sometimes installs toilets and hand-washing facilities on the job (U3) (47.0%); while for positive values for dimension 2, that the company does not install toilets and hand-washing facilities on the job (U2) (14.3%), and for negative values, that the company sometimes installs toilets and hand-washing facilities on the job (U3) (2.8%).

For variable V, the category that best expresses the positive values for dimension 1 is that the company installs urinals on the worksite (V1) (63.0%), and for negative values, that the company does not install urinals on the worksite (V2) (15.3%); while for positive values for dimension 2, that the company does not install urinals on the worksite (V2) (28.5%), and for negative values, that the company sometimes installs urinals on the worksite (V3) (18.6%). For variable X, the category that best accounts for positive values for dimension 1 is that the company installs changing rooms on the worksite (X1) (57.9%), and for negative values, that the company sometimes installs changing rooms on the worksite (X3) (14.7%); while for positive values for dimension 2, that the company does not install changing rooms on the worksite (X2) (18.0%), and for negative values, that the company sometimes installs changing rooms on the worksite (X3) (9.2%).

For variable Y, the category that best explains the positive values for dimension 1 is that the company installs a lunch area on the worksite (Y1) (53.5%), and for negative values that the company does not install a lunch area on the worksite (Y2) (18.6%); while for positive values for dimension 2, that the company does not install a lunch area on the worksite (Y2) (28.4%), and for negative values, that the company sometimes installs a lunch area on the worksite (Y3) (36.3%). For variable Z, the category that best expresses the positive values for dimension 1 is that the company indicates emergency routes and exits on the worksite (Z1) (60.5%), and for negative values, that the company does not indicate emergency routes and exits on the worksite (Z2) (16.9%); while for positive values for dimension 2, that the company does not indicate emergency routes and exits on the worksite (Z2) (29.7%), and for negative values, that the company sometimes indicates emergency routes and exits on the worksite (Z3) (25.9%).

For the variable ZZ, the category that best accounts for positive values in dimension 1 is that the company makes exclusive unloading zones available on the worksite (ZZ1) (63.3%), and for negative values, that the company sometimes makes exclusive unloading zones available on the worksite (ZZ3) (22.9%); while for positive values for dimension 2, that the company does not make exclusive unloading zones available on the worksite (ZZ2) (7.7%), and for negative values, that the company sometimes makes exclusive unloading zones available on the worksite (ZZ3) (16.7%). For variable II, the category that best accounts for positive values for dimension 1 is that the company installs safety networks on the worksite (II1) (57.2%), and for negative values, that the company does not install safety networks on the worksite (II2) (34.8%); while for positive values for dimension 2, that the company does install safety networks on the worksite (II1) (6.0%), and for negative values, that the company sometimes installs safety networks on the worksite (II3) (7.5%).

For variable KK, the category that best explains the positive values for dimension 1 is that the company considers aspects of ventilation on the worksite (KK1) (59.7%), and for negative values, that the company does not consider aspects of ventilation on the worksite (KK2) (43.0%); while for positive values for dimension 2, that the company does consider aspects of ventilation on the worksite (KK1) (4.0%), and for negative values, that the company sometimes considers aspects of ventilation on the worksite (KK3) (10.6%). For variable LL, the category that best expresses the positive values for dimension 1 is that the company considers aspects of noise protection on the worksite (LL1) (40.7%), and for negative values, that the company does not consider aspects of noise protection on the worksite (LL2) (41.2%); while for positive values for dimension 2, that the company does consider aspects of noise protection on the worksite (LL1) (5.6%), and for negative values, that the company sometimes considers aspects of noise protection on the worksite (LL3) (2.1%).

For variable SS, the category that best accounts for positive values for dimension 1 is that the company develops a Health and Safety Plan to execute work when working as the contractor (SS1) (20.4%), and for negative values, that the company does not develop a Health and Safety Plan to execute work when working as the contractor (SS2) (23.2%); while for positive values for dimension 2, that the company does not develop a Health and Safety Plan to execute work when working as the contractor (SS1) (17.2%), and for negative values, that the company sometimes develops a Health and Safety Plan to execute work when working as the contractor (SS3) (28.1%).

For variable TT, the category that best explains the positive values for dimension 1 is that the company develops a Health and Safety Plan to execute work when working as the contractor, based on the Health and Safety Study, which requires risk assessment and meetings being held with subcontractors (TT9) (4.1%), and for negative values, that the company prepares a Health and Safety Plan to execute work based on other indeterminate things when working as the contractor (TT4) (1.5%); while for positive values for dimension 2, that the company prepares a Health and Safety Plan to execute work when working as the contractor based on nothing (TT5) (0.3%), and for negative values, that the company prepares a Health and Safety Plan to execute work when working as the contractor, based on the Health and Safety Study, which requires risk assessment and meetings being held with subcontractors (TT9) (10.8%).

For variable AAA, the category that best expresses the positive values for dimension 1 is that the company holds health and safety meetings with the subcontractors to coordinate the work when working as the contractor (AAA1) (20.3%), and for negative values, that the company does not hold health and safety meetings with the subcontractors to coordinate the work when working as the contractor (AAA2) (23.2%); while for positive values for dimension 2, that the company does not hold health and safety meetings with the subcontractors to coordinate the work when working as the contractor (AAA2) (5.6%), and for negative values, that the company does hold health and safety meetings with the subcontractors to coordinate the work when working as the contractor (AAA1) (16.0%).

For variable EEE, the category that best accounts for positive values for dimension 1 is that the company has a high awareness (EEE3) of suffering accidents in its construction processes (3.6%), and for negative values, that the company has a low awareness (EEE1) of suffering accidents in its construction processes (0.6%); while for positive values for dimension 2, that the company has a medium awareness (EEE2) of suffering accidents in its construction processes (2.1%), and for negative values, that the company has an awareness of suffering accidents in its construction processes that is both high (EEE3) (0.2%) and low (EEE1) (0.2%).

#### 3.3.5. Object Scoring (Companies)

**Figure 3:**
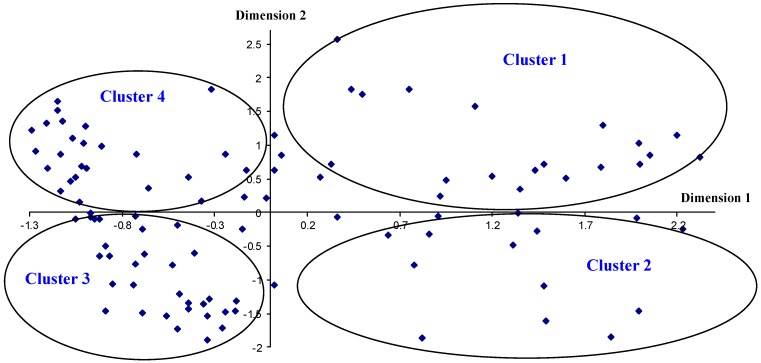
Factorial plane of the object scoring (companies).

Finally, the MCA allows one to represent a factorial plane of the objects (companies) using each of their scores in each of the two dimensions (Figure 3). In this representation, one can observe how the companies in the sample are grouped into 4 clusters with homogenous characteristics. Cluster 1 presents positive scores in both dimensions, cluster 2 presents positive scores for dimension 1 and negative for dimension 2, cluster 3 presents negative scores in both dimensions and cluster 4 presents negative scores in dimension 1 and positive in dimension 2.

## 4. Discussion

### 4.1. Study Limitations

For the characterization of workplace risk prevention in Guatemalan construction companies, a sample was considered containing 5.1% of the Prequalified Companies in the census from the Guatemalan Ministry of Communications, Infrastructure and Housing in operation as of December 2015 [49]; thus, the present study is an estimation based on the companies in the sample, but this might be different for other companies.

### 4.2. Descriptive Analysis of the Variables

#### 4.2.1. Characterization of the General Company Variables

Most of the construction companies in Guatemala (52.0%) dedicate their activity simultaneously to civil engineering, building construction and other specialized construction, while 21.0% only participate in civil engineering and building construction (a value below the 38% of companies in the construction sector of Andalusia, Spain, that are dedicated to both activities [47]), 13% only to building construction (a value below the 32% of companies in the Andalusian construction sector that are dedicated to both activities [47]), and 11.0% only to civil engineering (a value below 16% of companies in the Andalusian construction sector that are dedicated to both activities [47]). Regarding the Guatemalan departments (regional areas) where this activity takes place, their distribution varies greatly across the country: 26% of companies work all over the country, 17% in the Department of Guatemala REGION (I), another 17% in Departments of Guatemala REGION (I) together with Sacatepéquez, Escuintla, Chimaltenango, REGION IV, and 8% in the departments of Quetzaltenango, San Marcos, Totonicapán, Suchitepéquez, Retalhuleu, Sololá, REGION VI (see Figure 1). These data agree with the INE (Instituto Nacional de Estadística) statistics [49], which estimate that more than 50.0% of construction companies carry out activity in the Department of Guatemala.

Guatemalan construction companies have an average annual turnover (C) of 1.29 million euros (s.d. 6.29) such that 23.0% turnover between 0.3 and 0.5 million euros, 21.8% turnover between 0.1 and 0.3 million euros and 17.2% turn over less than 0.1 million euros. The average turnover obtained almost coincides with the average annual turnover of greenhouse construction companies in south-eastern Spain, which has been quantified at 1.56 million euros [48], such that 50% of these companies turnover less than one million euros and 30% turn over more than 2 million euros. Likewise, for construction companies as a whole in Andalusia, 29.0% of them turn over between 0.5 and 1 million euros [47]. The average number of company office workers (D) is 7.3 (s.d. 15.06), such that 61.2% have less than 6 office workers, 30.6% have between 6 and 10, 6.1% have between 11 and 20 and 2.1% have more than 20. The average number of on-site workers (E) is 81.1 (s.d. 75.22), such that 11.3% are microcompanies; that is to say, they have less than 11 workers (a very similar percentage to companies in the Andalusian construction sector as a whole, which is 14.0% [47]), and very far from the 50% of microcompanies in the greenhouse construction sector in south-eastern Spain [48]; while 39.2% are small companies, having between 11 and 50 workers, which is somewhat less than the small construction companies in Andalusia, which is 54% [47]. Finally, in Guatemala, the rest of the companies (49.5%) are medium size, such that 17.5% have between 51 and 100 workers and 13.4% between 101 and 150 workers.

The average number of work crews per year (F) that make up Guatemalan construction companies is 6.8 (s.d. 5.55), a value above the 4.4 (s.d. 2.59) that is the average number of work crews making up greenhouse construction companies in south-eastern Spain [48]. This is logical given that the difference in the number of microcompanies that exist between the two sectors. Of Guatemalan construction companies, 41.7% have between 4 and 6 work crews, 22.9% between 7 and 10, and 22.9% with less than 4 work crews, while in the greenhouse construction sector in south-eastern Spain, 40.0% have less than 5 work crews [48]. Regarding the average number of years that the Guatemalan construction companies have carried out on-site works (G), this amounts to 2.7 years (s.d. 0.55), such that 49.0% have been working for between 11 and 20 years, 32.7% for less than 11 years and 15.3% for between 21 and 30 years.

Finally, 47.5% of Guatemalan construction companies work on jobs solely as contractors, while in the Andalusian construction sector, it is only 36.0% [47], and in the greenhouse construction sector in south-eastern Spain [48], it is 80.0–25.2% as contractors and subcontractors, 16.0% in the Andalusian construction sector [47], 20.0% in the greenhouse construction sector of south-eastern Spain [48], and 16.2% as developer, contractor and subcontractor.

#### 4.2.2. Characteristics of the Prevention Activities and Health and Safety Management in the Companies and on the Building Sites

Currently, to achieve fewer accidents on construction sites, there has been for some time techniques for the prevention and management of workplace risks in construction companies via the design process [14,16,32,34,35]. Accordingly, 42.0% of companies reported that they carry out a prevention plan before starting a construction job and 43.0% do so sometimes. With regard to preventative measures adopted on site, 28.0% reported that they practice those of personal protection, collective protection and signaling at the same time, and another 28.0% only those of personal protection and signaling at the same time, with 19.0% that only practice those of personal protection. In comparison, the totality of companies in the greenhouse construction sector of south-eastern Spain confirmed that they adopt personal protection measures for workers but do not adopt those of collective protection and do not carry out on-site signaling [48]. 25.0% of the Guatemalan construction companies that reported their workers adopting safety measures, do so for head, eye, hands and feet protection, and 10.0% for head, ear, eye, hand, nose, feet and face protection; while less than 56.0% reported that they protect their heads with safety helmets, the remaining 44.0% do not use them to protect their heads, a value that is significantly below that reported by Pérez-Alonso and collaborators for workers in the greenhouse construction sector in south-eastern Spain [48], which stated that 100.0% of workers do not use safety helmets; these data confirm the results obtained by Tam and collaborators for the construction sector in China [26], in which the workers in the sample reported that the safety helmet was not convenient for many operations. Likewise, in China, Chia-Fen and collaborators determined that the smallest construction companies presented a higher incidence of falls from heights owing to a lack of experience, of training and the generalized practice of not using personal or collective protection equipment [51]. In this regard, various authors have reported that not using personal or collective protection equipment is a major source of accidents in the construction sector [18,21,22]. Regarding the work clothing used by workers in Guatemalan construction, 37.0% of companies reported that they use T shirts and hi-viz jackets, 16.0% T shirts, hi-viz jackets, and a uniform or canvas trousers, 14.0% use only hi-viz jackets and 12.0% T shirts and a uniform or canvas trousers. Therefore, on many occasions, the work clothing is inadequate, as described by Pérez-Alonso and collaborators in the greenhouse construction sector in the south-east of Spain [48]. Consequently, in these circumstances, unsatisfactory environmental conditions can be produced in the workplace, as confirmed by various authors, in such a way that small companies present worse workplace conditions and a greater risk of worker accidents [52,53,54,55,56].

Regarding health and safety training for the workers, Cheng and collaborators reported that it is fundamental to avoid accidents on the worksite [1], but that on many occasions the construction companies do not provide it sufficiently to reduce costs. In addition, not only this, there are companies, mostly microcompanies, that assume that the workers must know themselves what to do to avoid accidents in the building procedures that need to be developed on site [57]. In Guatemalan construction companies, 42.4% give training, 53.6% reported that they sometimes do, and the remaining 4% reported that they provide none; this results in a lack of safety [26], which contrasts with the current tendency where health and safety in the workplace is taught through design at the degree and postgraduate levels at universities [33]. However, in the greenhouse construction companies of south-eastern Spain [48], only 50.0% carry out such training prior to work, a lower percentage to that determined by Calderón for Andalusian construction companies (Spain) [47], which was 62.5%. Regarding the right that workers have to a medical assessment, only 12.4% of the sampled companies provide this, 15.4% do so sometimes, whereas 72.2% reported that they do not do so; these values differ greatly from the 70.0% of greenhouse companies in south-eastern Spain [48] and the 86.0% of Andalusian construction companies that provide it [47].

On the other hand, employers are obliged to perform an initial risk assessment as well as other periodical ones, as appropriate; hence 43.0% of sampled companies reported that they risk assess before starting the job, 22.0% before and during the job, and 16.0% during the job, and 13% before, during and at the end of the job, and only 3.0% reported that they never do so; this last value is very small compared to the 30.0% reported by Pérez-Alonso and collaborators for greenhouse construction in the south-east of Spain [48]. Regarding how safety and risk control is carried out on the job, 56.6% of sampled companies only carry it out using a supervisor, 13.1% using a supervisor and a safety foreman, and 4.0% reported that they do not do so. The frequency with which the sampled companies verify the on-site risks and safety is, 22.0% at the start of a job, 19.0% at the beginning and the end of a job, and 19.0% at the start and daily. Accordingly, Goh and collaborators reported that exhaustive maintenance has to be performed along with periodic safety inspections [27], continuous safety training and the development of a plan to investigate accidents to prevent future injury; consequently, there are companies that not only possess such controls but that point them out as much as possible, in order to give them greater relevance among companies that work transparently and wish to gain greater legitimacy [58].

Continuing with the sampled companies, 28.0% reported that there is an entity that obliges them to control risks on site, while another 28.0% reported that there is not. When this entity exists, 59.0% report that it is the contractor or developer, 18.1% that it is the Ministry of Work and 8.2% that it is the Social Security Institute. Safety is a process that considers the use of all types of equipment and techniques to systematically guarantee the well-being of the workers [23,24,25]; therefore, the control of equipment and machinery by the company within the prevention management framework is important. Therefore, for companies in the Guatemalan construction sector, 18.0% reported that they do have an operation manual available for tools and equipment, 58.0% reported that they sometimes have one available for some of them, and 24.0% reported that one does not exist. 45.0% have a tool and equipment inventory available while 20.0% do not. Likewise, 31.0% give training on tool and equipment use while 23.0% reported that they do not. With respect to the control of equipment handed to the workers, 34.3% of sampled companies do control the equipment provided with a loading and unloading card while 35.4% do not. 22.2% of companies make an operation manual for their machinery available while 13.1% do not. In addition, for handling this machinery, 39.4% of companies reported that they do give training on how to use it while 13.1% do not. Similarly, 64.0% of companies reported that they make first-aid equipment available as well as personal training on how to use it while 30.0% said they do so sometimes, and 6.0% said that they do not. As a comparison to all the prevention management indicators detailed in the present paragraph, in greenhouse construction companies in south-eastern Spain [48], 50.0% make a work equipment identification list available while the other 50.0% do not; and regarding the existence of an equipment maintenance log, 60.0% of these companies make them available.

Recently, passive safety indicators have begun to be implemented that formulate specific programs and political strategies; for example, in Tennessee (USA), the most popular safety indicators proposed were housekeeping, the use of PPE and substance abuse [59], affirming moreover, that the largest companies are most likely to use the safety indicators than are smaller companies. In this context, and in relation to on-site hygiene, health, and safety measures in Guatemalan construction companies, they indicated that 51.0% of them make a place available on site to dispose of waste or rubbish, 42.0% sometimes do and 7.0% do not. 49.0% of the companies indicated that they carry out general site cleaning daily, 41.8% do so once a week and 3.3% do so on demand. Regarding the toilet facilities provided on site for the workers, 40.0% indicated that they provide toilet paper and bars of soap, or soap powder, 18.6% provide toilet paper, bars of soap or soap powder, and paper or cloth towels, while 17.1% only provide toilet paper and 8.6% only provide bars of soap or soap powder. Likewise, 42.3% install on-site toilets, 46.4% indicated that sometimes they do so and 11.3% say that they do not. Regarding whether they install urinals, 40.4% say they do not, 30.9% indicated that they do and 28.7% said they do so sometimes. Only 20.8% indicated that they install emergency showers on site, 47.9% do not and 31.3% said they do so sometimes. Regarding installing on-site changing rooms for workers, only 26.3% indicated that they do, while 42.1% do not, and 31.6% indicated that they do so sometimes. Conversely, 40.0% of sampled companies plan specific zones for offloading on site, while 17.0% do not and 43.0% do so sometimes. Only 25.0% of companies indicated that they install on-site safety networks, while 40.0% do not. As to whether the companies consider on-site lighting, 37.4% indicated that they do, while 10.1% said they do not. Concerning on-site ventilation, 56.6% said they do not provide it while only 24.2% said they do. With respect to on-site noise protection, only 16.3% indicated that they provide protection while 58.2% said they do not. Likewise, 41.7% indicated that they install electric cabling protection on site while 20.8% said they do not.

Regarding prevention management related to actions and communications in case of on-site accidents, one must emphasize that 92.0% of companies indicated that they do have procedures available in the case of on-site accidents, with the remaining 8.0% do not have any. Regarding where the victim of an accident is treated if an accident occurs, 25.8% of sampled companies indicated that it is the infirmary, health center, public hospital, Guatemalan Social Security Institute, or private hospital closest to the worksite, with 9.7% stating the nearest infirmary, public hospital, or Guatemalan Social Security Institute. 42.3% of the companies indicated that they do not communicate the accidents suffered on site with any entity, while only 13.4% said that they do, and of these, if they do so, 71.4% contact the contractor or the developer, while 12.2% contact the contractor or developer and the Guatemalan Social Security Institute, with 6.1% only notifying the Guatemalan Social Security Institute. Finally, with respect to the awareness of accident risk presented by the Guatemalan construction companies, they indicated that 63.0% consider there is a low probability of suffering worksite accidents, 33% considered it a medium probability and the remaining 4% considered it a high risk. This large percentage of companies that had a low probability perception of on-site accident risk is a clear indication of the limited risk management and training in those companies, as cited by Rodríguez-Garzón and collaborators [60,61], who stated that the companies presuppose that risk awareness should be something innate to the worker.

To summarize the issue at hand, one must point out that a high percentage of Guatemalan construction companies (72.2%) do not perform medical assessments on their workers, and also a medium percentage of companies do not practice tool, machinery and equipment control, nor give specific training for operating the type of apparatus used on worksites, all of which demonstrates insufficient safety management [27,47,48], owing to a company lacking a safety policy, that in turn, can lead to a higher level of accidents [26,27,28,30,47,48,54].

#### 4.2.3. Characteristics of the Health and Safety Activities That Contractors Take Part in on the Worksite

According to several authors [5,16,33,47,48], building work ought to be carried out following a technical project prepared by a competent technician, which includes a Health and Safety Study for the work to be done—this then becomes a worksite Health and Safety Plan as soon as work begins, which the contractor should propose to the works management and the Health and Safety Coordinator. For those Guatemalan construction companies that said they operated as contractors, only 13.4% reported that they prepare this Health and Safety Plan, 34.2% do not prepare one and 52.4% do so sometimes; data which differ greatly from those reported by Calderón regarding Andalusian construction companies (Spain) [47], in which 95.0% said they prepare a Health and Safety Plan, while only 3.3% do not. Nonetheless, the Guatemalan data are better than those reported by Pérez-Alonso and collaborators [48], regarding greenhouse construction companies in south-eastern Spain, in which none of the companies prepared such a plan.

In addition, the comparison of the study results with those obtained by Calderón for the Andalusian construction industry (Spain) [47] continues. The last results are expressed in parentheses. When Guatemalan construction companies that work as contractors were asked how they prepare the worksite Health and Safety Plan, 38.6% reported they do so through prior meetings with the subcontractors, 12.3% base it on the Health and Safety Study (86.7%), and 7.0% base it on the Health and Safety Study as well as asking for a risk assessment from the subcontractors with prior meetings, while 5.2% base it on the Health and Safety Study and prior meetings with the subcontractors. Regarding whether the stipulations proposed by the Health and Safety Plan are complied with on the job, only 20.3% reported that they always are (71.0%), while 17.6% never, and 62.2% only partially. Regarding whether the contractors send a copy of the Health and Safety Plan to the subcontractors, 14.9% reported that they do (93.5%) while 41.9% reported that they do not and 43.2% that they do so only sometimes. Likewise, 42.1% of companies who operated as contractors indicated that they know the worksite Health and Safety Coordinator, while 57.9% said they do not; this contrasts with the 100% that stated that they know them for construction companies in Andalusia [47]. Regarding the frequency of visits by the Health and Safety Coordinator to the worksite, 21.1% of sampled companies who work as contractors reported that this is once or twice a week (39.3%), 15.8% said it is once a fortnight (25.0%), 5.3% once a week and 7.9% indicated that they do not know (10.7%). Regarding who pays the Health and Safety Coordinator’s fee, 44.9% of the sampled companies who act as the contractor reported that they pay themselves (43.7%), 32.7% reported that the developer pays and 22.4% said it is the contractor and the developer. Likewise, 16.2% of sampled companies reported that the developer evaluates the health and safety measures that the contractor company will undertake when awarding the work (28.1%), while 29.7% reported that the developer does not evaluate such measures and 54.1% that they sometimes do so (43.8%). Regarding whether the company acting as the contractor hold safety meetings with the subcontractors to disclose the progress and incidents of on-site safety conditions, only 15.2% of companies indicated that they do (41.4%), while 46.8% reported that they do not (6.9%), and the remaining 38.0% do so sometimes (51.7%). With respect to whether it would be of interest to classify contractor companies based on the quality and management of workplace risk prevention in order to improve on-site safety conditions, 51.8% of the sampled contractor companies indicated that such a classification is necessary (71.9%), while only 2.5% said that it would not be (9.3%), and the remaining 45.7% said that it probably would be necessary (18.8%). Finally, 10.0% of the sampled contractor companies indicated that when they subcontract a job, they evaluate the subcontractor based on their level of safety (32.3%), while 42.5% said they do not (19.3%), and 47.5% that they do so sometimes (48.4%).

The low percentages of contractor companies that said they prepare a Health and Safety Plan (13.4%) and comply on site with the stipulations it contains (20.3%) reveals a lack of safety [5,8,16,26,47,48], which, in turn, results in a greater incidence of accidents [5,8,16,28,47,48]. All of this is due to the absence of a clear safety policy in Guatemalan construction companies, and as indicated by Hasle and collaborators [54], the companies do not consider safety a priority.

### 4.3. MCA

Interpreting the factorial planes in Figure 2 and Figure 3, one can observe the correspondences between the categories of variables and thus the characterization of each of the 4 clusters of specific companies. Both companies in cluster 1 and in cluster 2 have the categories that nullify, or practically nullify the quantification in dimension 2 of the following variables: they do develop a prevention plan at the start of the work (I1); they give workplace prevention training to the workers (L1); they install toilets (U1), urinals (V1), emergency showers (W1), changing rooms (X1), on-site emergency routes (Z1) and they do know an entity that obliges them to control on-site health and safety (GG1). On the other hand, companies in cluster 1 are characterized by presenting all the categories of variables with positive quantifications in dimensions 1 and 2, the most significant being that the companies only work on civil engineering construction (A1), or only on building construction (A2), or both activities at the same time (A4); that present a medium awareness of suffering some worksite accidents (EEE2); that operate on jobs solely as the developer (H1), or solely as the subcontractor (H3), or at the same time as the developer, contractor and subcontractor (H7); that their turnover is less than 0.1 million euros (C1); that the number of on-site workers is less than 11 (E1) or between 11 and 50 (E2); that the number of work crews is less than 4 (F1); that they adopt only collective protection as on-site preventative measures (J1), or on-site collective protection and signaling (J11), or personal protection and color coding (J12), or collective protection and personal protection (J13); that they perform medical assessments on the workers (M1); that they install an on-site lunch area (Y1); that they establish a specific on-site unloading zone (ZZ1); that they perform on-site risk assessment during the work (P4), at the end of the work (P8), or never (P6), or that they do not respond to the question (P7); that they carry out an equipment inventory (BB1); that they give equipment-handling training (CC1); that they place safety networks on site (II1); that they use on-site lighting when necessary (JJ1); that they use on-site ventilation when necessary (KK1); and that they use on-site acoustic protection (LL1).

Companies in cluster 2 are characterized by presenting all the categories of variables with positive quantifications in dimension 1 but negative in dimension 2. The most significant are that the company works on site as the developer or as the contractor (H4); that it turns over between 1 and 2 million euros (C6), or between 2 and 10 million euros (C7) or more than 10 million euros (C8); that the number of on-site workers is between 151 and 200 (E5) or more than 200 (E6); that the number of work crews is more than 20 (F5); that they adopt collective protection, personal protection, color coding and signaling as on-site prevention measures (J8), or as above but without collective protection (J6); that they sometimes carry out medical assessments on the workers (M3); that they carry out on-site risk assessment before and during a job (P2), or before, during and at the end of a job (P5); that they sometimes have acoustic protection on site (LL3); that they prepare a Health and Safety Plan (SS1); that they prepare the Health and Safety Plan based on a risk assessment requested only from the subcontractors (TT2) or on the Health and Safety Study with prior meetings held with the subcontractors (TT6), or that they request a risk assessment from the subcontractors and hold prior meetings with the subcontractors (TT8), or they base it on the Health and Safety Study, request a risk assessment from the subcontractors as well as prior meetings with the subcontractors (TT9), or on the Health and Safety Study, request a risk assessment from the subcontractors as well as prior meetings with the subcontractors and others (TT10), and that they know the Health and Safety Coordinator for the job (WW1); and finally that they present a high awareness of suffering some worksite accidents (EEE3).

The companies in cluster 3 are characterized by presenting all the categories of the variables with negative quantifications in dimensions 1 and 2, the most significant are that the companies construct in the 3 activities, civil engineering, building construction and specialized construction (A7); that they work on jobs solely as contractors (H2); that they turn over between 0.5 and 0.7 million euros (C4) or between 0.7 and 1.0 million euros (C5); that the number of workers on site is between 101 and 150 (E4); that the number of work crews is between 7 and 10 (F3) or between 11 and 20 (F4); that as on-site preventative measures, they adopt either collective and personal protection and signaling (J5), or personal protection and on-site signaling (J7); that sometimes on site they install toilets (U3), urinals (V3), showers (W3), changing rooms (X3), a lunch area (Y3), emergency routes (Z3) and unloading zones (ZZ3); that they perform an on-site risk assessment during and at the end of the job (P3); that they sometimes carry out an equipment inventory (BB3); that they sometimes give equipment-handling training (CC3); that they sometimes know an entity that obliges them to control on-site health and safety (GG3); that they sometimes locate safety networks on site (II3); that they sometimes use lighting on site when it is necessary (JJ3); that sometimes they have ventilation on site when necessary (KK3); that they sometimes prepare an on-site Health and Safety Plan (SS3), that the Health and Safety Plan is based on the Health and Safety Study (TT1) or on others (TT4); that sometimes they hold health and safety meetings with the subcontractors when they act as contractors on the job (AAA3).

The companies in cluster 4 are characterized as presenting all the categories of variables with negative quantifications in dimension 1 and positive quantifications in dimension 2, the most significant are that they only construct specialized buildings (A3), or civil engineering and specialized constructions (A5); that they work on jobs solely as contractors and subcontractors (H6); that they turn over between 0.1 and 0.3 million euros (C2), or between 0.3 and 0.5 million euros (C3); that the number of on-site worker is between 51 and 100 (E3); that the number of work crews is between 4 and 6 (F2); that they do not prepare an on-site prevention plan (I2); that they adopt on-site preventative measures only for personal protection (J2); that they do not give workplace prevention training to their workers (L2); that they do not perform medical assessments on their workers (M2); that they do not install toilets on site (U2), or urinals (V2), or showers (W2), or changing rooms (X2), or a lunch area (Y2), or emergency routes (Z2), or unloading zones (ZZ2); that they perform a risk assessment before the start of the job (P1); that they do not perform an equipment inventory (BB2); that they do not give equipment-handling training (CC2); that they do not know an entity that obliges them to control on-site health and safety (GG2); that they do not place safety networks on site (II2); that they do not use on-site lighting (JJ2); that they do not use ventilation on site when it is necessary (KK2); that they do not prepare an on-site Health and Safety Plan (SS2); that they base their Health and Safety Plan on nothing (TT5); that they do not know the on-site Health and Safety Coordinator (WW2), and that they do not hold health and safety meetings with the subcontractors when they act as contractors on the job (AAA2).

Likewise, both the companies in cluster 3 and those in cluster 4 present the categories that make quantification null in dimension 2, or practically null, for the following variables: that they sometimes give workplace prevention training to their workers (L3); that they do not practice on-site acoustic protection (LL2), and finally, that they present a low awareness of suffering any workplace accidents (EEE3).

Of the characteristics described in the four clusters regarding Guatemalan construction companies, one can clearly observe that the larger companies (higher turnover, more on-site workers and more work crews) are those that adopt better preventative measures and Health and Safety Management in the company and on site in the different activity areas [26,28,30,48,51,57,60,61], so that the largest correlate with a high awareness of suffering on-site accidents while those of medium size have a lower awareness of suffering workplace accidents, and they consider it to be medium, as other authors have confirmed about the construction sector in other countries, while the smaller companies present worse physical conditions in the workplace and a higher risk of accidents for their workers [18,48,52,53,54,55,56,57,60,61].

### 4.4. Recommendations

According to everything discussed, the following recommendations are proposed to improve the accidents prevention in the companies of the Guatemalan construction industry, especially the small and medium ones:

- Make a great effort to increase training in matters of safety and health prevention of their managers and workers, as well as the entrepreneurs themselves.
- Adopt as a priority, perform medical examinations to their workers, both when joining the company, and through their professional career in it.
- Implement a mandatory risk assessment in all phases of the construction works.
- Establish operating manuals for tools, equipment, and machinery, carrying out an inventory and providing training in their management.
- Provide work clothes that are better suited to the task developed by the worker.
- Communicate compulsorily the accidents suffered in the company with the periodicity established by the competent authority.
- Increase the use and compliance of Safety and Health Plans in the construction works.
- Promote the participation of safety and health coordinators in the design, planning and execution of the construction works.

Likewise, it would be advisable that the competent labor administration of Guatemala:

- Complement the existing labor legislation for the construction industry, to ensure adequate prevention and risk management in these companies.
- Promote educational plans for all professionals involved in construction works that incorporate a minimum of mandatory training in safety and health.
- Promote economic aid and/or tax incentives to companies, so that they adopt appropriate safety and health practices, checking and certifying that they perform it.

## 5. Conclusions

The present study investigated the characterization of the occupational risk prevention in the Guatemalan construction industry, correlating parameters of the prevention activity and occupational risk management with structural and organizational parameters of the companies. It was carried out using the simple random sampling technique and a questionnaire developed for it.

As a whole, the companies studied in the Guatemalan construction sector are characterized as operating mostly (52.0%) in civil engineering work, building construction and other specialized construction, and that they work mostly as contractors (47.5%). Likewise, they are characterized as being medium-sized companies, with an average of 81.1 on-site workers per year, having an average of 6.8 on-site work crews annually and an average annual turnover of 1.29 million euros.

By means of the multivariate technique of MCA, it has been possible to group companies in the Guatemalan construction industry into 4 clusters, with homogeneous characteristics, so that the larger construction companies (larger turnover, more on-site workers, and more work crews) adopt better preventative measures and Health and Safety Management, both on site and in the company as a whole, in such a way that the largest are correlated with a high awareness of suffering on-site accidents, while those of medium size have a medium awareness. In contrast, companies with a small number of workers more poorly manage workplace risk prevention, with a low awareness of accident risk, which is typical of a low level of prevention management.

Recommendations have been proposed to construction companies and the competent labor administration in Guatemala to improve accident prevention.

## Appendix A

**Table A1:** Nomenclature and frequency of the categories of general company variables.

Variable/Category Codes	Variables/Category of Variables	Frequency (%)
**A**	**Construction activity**	100.0
A1	Only civil engineering	11.0
A2	Only building construction	13.0
A3	Only specialized construction	2.0
A4	Civil engineering and building construction	21.0
A5	Civil engineering and specialized construction	1.0
A6	Construction of building and specialized construction	0.0
A7	Civil engineering, building and specialized construction	52.0
**B**	**Guatemalan departments where the work is carried out ^1^**	100.0
B4	Throughout the country	26.0
B9	Quetzaltenango, San Marcos, Totonicapán, Suchitepéquez, Retalhuleu, Sololá, REGION VI	8.0
B13	Guatemala REGION I, Sacatepéquez, Escuintla, Chimaltenango. REGION IV	17.0
B18	Guatemala REGION I	17.0
**C**	**Annual company turnover (€)**	87.0
C1	<100,000	17.2
C2	100,001–300,000	21.8
C3	300,001–500,000	23.0
C4	500,001–700,000	12.6
C4	700,001–1,000,000	11.5
C6	1,000,001–2,000,000	8.0
C7	2,000,001–10,000,000	4.6
C8	>10,000,000	1.1
**D**	**Number of office workers in the company**	98.0
D1	<6	61.2
D2	6–10	30.6
D3	11–20	6.1
D4	>20	2.1
**E**	**Number of on-site workers in the company**	97.0
E1	<11	11.3
E2	11–50	39.2
E3	51–100	17.5
E4	101–150	13.4
E5	151–200	11.3
E6	>200	7.2
**F**	**Number of work crews annually**	96.0
F1	<4	22.9
F2	4–6	41.7
F3	7–10	22.9
F4	11–20	10.4
F5	>20	2.1
**G**	**Years working on site**	98.0
G1	0–10	32.7
G2	11–20	49.0
G3	21–30	15.3
G4	31–40	2.0
G5	>40	1.0
**H**	**Work on the job**	99.0
H1	Only as the developer	2.0
H2	Only as the contractor	47.5
H3	Only as the subcontractor	2.0
H4	As developer and contractor	7.1
H5	As developer and subcontractor	0.0
H6	As contractor and subcontractor	25.2
H7	As developer, contractor, and subcontractor	16.2

^1^ The other categories were not included as many were not representative.

**Table A2:** Nomenclature and frequency of the categories of variables for Prevention Activities and Health and Safety Management in the company and on site (1st part).

Variable/Category Codes	Variables/Category of Variables	Frequency (%)
**I**	**Execute prevention plan before starting the job**	100.0
I1	Yes	42.0
I2	No	15.0
I3	Sometimes	43.0
**J**	**Preventative measures adopted on site ^1^**	100.0
J1	Only collective protection	3.0
J2	Only personal protection	19.0
J5	Collective and personal protection plus signaling	28.0
J6	Personal protection, color coding plus signaling	3.0
J7	Personal protection plus signaling	28.0
J8	Collective and personal protection, color coding plus signaling	14.0
J11	Collective protection and signaling	1.0
J12	Personal protection and color coding	2.0
J13	Collective and personal protection	2.0
**K**	**Types of personal protection adopted on site ^1^**	100.0
K13	Head, ear, eye, hand, nose, feet, and face protection as well as fall protection (harness)	10.0
K18	Head, eye, hand, and feet protection as well as fall protection (harness)	8.0
K19	Head, eye, ear, hand, nose, and feet protection as well as fall protection (harness)	6.0
K22	Head, eye, ear, hand, nose, feet, face, and skin protection as well as fall protection (harness)	7.0
K29	Head, ear, eye, hand, nose, feet, and face protection as well as fall protection (harness)	25.0
**L**	**Training given to workers on health and safety**	99.0
L1	Yes	42.4
L2	No	4.0
L3	Sometimes	53.6
**M**	**Medical assessments carried out on the workers**	97.0
M1	Yes	12.4
M2	No	72.2
M3	Sometimes	15.4
**N**	**Modality of on-site safety and risk control ^1^**	99.0
N1	Supervisor	56.6
N2	Employer assumes responsibility for prevention	6.1
N3	Supervisor and safety manager	13.1
N7	No modality	4.0
N8	Other modalities	3.0
N9	Safety manager	3.0
N14	Supervisor, safety manager, design group or commission and in-house permanent service	3.0
**O**	**How often are on-site risks and safety verified**	100.0
O1	At the beginning	22.0
O2	Daily	11.0
O3	Weekly	7.0
O4	At the end	3.0
O5	Never	7.0
O6	At the beginning and at the end	19.0
O7	At the beginning and weekly	11.0
O8	At the beginning and daily	19.0
O9	At the beginning, weekly and at the end	1.0
**P**	**When is the on-site risk assessment carried out**	100.0
P1	Before starting the work	43.0
P2	Before starting and during the work	22.0
P3	During and at the end of the work	1.0
P4	During the work	16.0
P5	Before starting, during and at the end of the work	13.0
P6	Never	3.0
P7	Before starting and at the end of the work	1.0
P8	At the end	1.0

^1^ The other categories were not included as many were not representative.

**Table A3:** Nomenclature and frequency of the categories of variables for Prevention Activities and Health and Safety Management in the company and on site (2nd part).

Variable/Category Codes	Variables/Category of Variables	Frequency (%)
**Q**	**First-aid equipment available and personnel trained to use it**	100.0
Q1	Yes	64.0
Q2	No	6.0
Q3	Sometimes	30.0
**R**	**A place is available on site to discard waste and rubbish**	100.0
R1	Yes	51.0
R2	No	7.0
R3	Sometimes	42.0
**S**	**Frequency of general cleaning carried out on site**	91.0
S1	Daily	54.9
S2	Once a week	41.8
S3	On demand	3.3
**T**	**Toilet facilities provided on site ^1^**	70.0
T1	Toilet paper and bars of soap or soap powder	40.0
T2	Bars of soap or soap powder	8.6
T5	Toilet paper	17.1
T7	Toilet paper, bars of soap or soap powder, and paper or cloth towels	18.6
T9	Toilet paper, bars of soap or soap powder, paper or cloth towels, and sponge or brush for the skin	7.1
**U**	**Toilets installed on site**	97.0
U1	Yes	42.3
U2	No	11.3
U3	Sometimes	46.4
**V**	**Urinals installed on site**	94.0
V1	Yes	30.9
V2	No	40.4
V3	Sometimes	28.7
**W**	**Emergency showers installed on site**	96.0
W1	Yes	20.8
W2	No	47.9
W3	Sometimes	31.3
**X**	**Changing rooms installed on site**	95.0
X1	Yes	26.3
X2	No	42.1
X3	Sometimes	31.6
**Y**	**Lunch area installed on site**	98.0
Y1	Yes	21.5
Y2	No	46.9
Y3	Sometimes	31.6
**Z**	**Emergency routes installed on site**	99.0
Z1	Yes	30.3
Z2	No	35.4
Z3	Sometimes	34.3
**ZZ**	**Zones designated specifically for unloading on site**	100.0
ZZ1	Yes	40.0
ZZ2	No	17.0
ZZ3	Sometimes	43.0

^1^ The other categories were not included as many were not representative.

**Table A4:** Nomenclature and frequency of the categories of variables for Prevention Activities and Health and Safety Management in the company and on site (3rd part).

Variable/Category Codes	Variables/Category of Variables	Frequency (%)
**AA**	**A tool and equipment operating manual exists**	100.0
AA1	Yes	18.0
AA2	No	24.0
AA3	Some	58.0
**BB**	**A tool and equipment inventory exists**	100.0
BB1	Yes	45.0
BB2	No	20.0
BB3	Sometimes	35.0
**CC**	**Tool and equipment-handling training is given**	100.0
CC1	Yes	31.0
CC2	No	23.0
CC3	Sometimes	46.0
**DD**	**The loading and unloading of provided equipment is controlled with a card**	99.0
DD1	Yes	34.3
DD2	No	35.4
DD3	Sometimes	30.3
**EE**	**A machinery operating manual exists**	99.0
EE1	Yes	22.2
EE2	No	13.1
EE3	Some	64.7
**FF**	**Machinery operation training is given**	99.0
FF1	Yes	39.4
FF2	No	13.1
FF3	Sometimes	47.5
**GG**	**Some entity exists that makes the control of on-site risks obligatory**	100.0
GG1	Yes	28.0
GG2	No	28.0
GG3	Sometimes	44.0
**HH**	**Which entity obliges the control of on-site risks**	61.0
HH1	Contractor or developer	59.0
HH2	The Social Security Institute and the Ministry of Work	1.6
HH3	Contractor or developer and the Ministry of Work	1.6
HH4	Contractor or developer and the Ministry of Work and the local authority	6.6
HH5	The Social Security Institute	8.2
HH6	The Ministry of Work and the local authority	3.3
HH7	The Ministry of Work	18.1
HH8	Contractor or developer and the Social Security Institute	1.6
**II**	**Safety networks located on site**	100.0
II1	Yes	25.0
II2	No	40.0
II3	Sometimes	35.0
**JJ**	**Lighting is considered on site**	99.0
JJ1	Yes	37.4
JJ2	No	10.1
JJ3	Sometimes	52.5
**KK**	**Ventilation is considered on site**	99.0
KK1	Yes	24.2
KK2	No	56.6
KK3	Sometimes	19.2
**LL**	**Protection against noise is considered on site**	98.0
LL1	Yes	16.3
LL2	No	58.2
LL3	Sometimes	25.5

**Table A5:** Nomenclature and frequency of the categories of variables for Prevention Activities and Health and Safety Management in the company and on site (4th part).

Variable/Category Codes	Variables/Category of Variables	Frequency (%)
**MM**	**Electric cable protection is installed on site**	96.0
MM1	Yes	41.7
MM2	No	20.8
MM3	Sometimes	37.5
**NN**	**Types of clothing worn by personnel on site**	100.0
NN1	T-shirt	2.0
NN2	Hi-viz jacket	14.0
NN3	No specific work clothes	1.0
NN4	Others (uniform or canvas trousers)	4.0
NN5	T-shirt and Hi-viz jacket	37.0
NN6	T-shirt and others (Uniform or canvas trousers)	12.0
NN7	T-shirt and Hi-viz jacket and others (uniform or canvas trousers)	16.0
NN8	T-shirt, Hi-viz jacket, work smock and others (uniform or canvas trousers)	6.0
NN9	Hi-viz jacket and others (uniform or canvas trousers)	3.0
NN10	Hi-viz jacket and work smock	2.0
NN11	T-shirt, Hi-viz jacket and work smock	2.0
NN12	Hi-viz jacket, work smock and others (uniform or canvas trousers)	1.0
**OO**	**An action protocol is available in the case of an on-site accident**	100.0
OO1	Yes	92.0
OO2	No	8.0
**PP**	**Where an on-site accident victim is treated ^1^**	93.0
PP3	The nearest Social Security Institute	6.5
PP7	The nearest infirmary, health center, public hospital, Social Security Institute, or private hospital/clinic	25.8
PP8	The nearest infirmary, health center, public hospital, or Social Security Institute	7.5
PP13	The nearest infirmary, public hospital, or Social Security Institute	9.7
PP14	The nearest health center, public hospital or Social Security Institute, or a private hospital/clinic	5.4
**QQ**	**Any entity contacted following accidents suffered on site**	97.0
QQ1	Yes	13.4
QQ2	No	42.3
QQ3	Sometimes	44.3
**RR**	**Entity or organization contacted after an accident has occurred**	49.0
RR1	Contractor or developer	71.4
RR3	Others	4.1
RR4	Contractor or developer and the Ministry of Work	12.2
RR5	Contractor, developer, and the Guatemalan Social Security Institute	6.1
RR6	The Guatemalan Social Security Institute	2.1
RR7	The Ministry of Work	4.1
**EEE**	**Company awareness of the risk of suffering on-site accidents**	100.0
EEE1	Low awareness of accident risk	63.0
EEE2	Medium awareness of accident risk	33.0
EEE3	High awareness of accident risk	4.0

^1^ The other categories were not included as many were not representative.

**Table A6:** Nomenclature and frequency of the categories of the health and safety variables for Contractor Companies.

Variable/Category Codes	Variables/Category of Variables	Frequency (%)
**SS**	**A Health and Safety Plan is prepared for the jobs carried out**	82.0
SS1	Yes	13.4
SS2	No	34.2
SS3	Sometimes	52.4
**TT**	**What the Health and Safety Plan is based upon ^1^**	57.0
TT1	A Health and Safety Study	12.3
TT2	A risk assessment is only requested from the subcontractor	3.5
TT3	Prior meetings with the subcontractor	38.6
TT4	Others	26.3
TT5	On nothing	3.5
TT6	A Health and Safety Study and prior meetings with the subcontractor	5.2
TT8	A risk assessment is requested from the subcontractor and prior meetings are held with them	1.8
TT9	A Health and Safety Study, a risk assessment requested from the subcontractor and prior meetings are held with them	7.0
TT10	A Health and Safety Study, a risk assessment requested from the subcontractor and prior meetings are held with them, and others	1.8
**UU**	**The stipulations of the Health and Safety Plan are complied with on site**	74.0
UU1	Always	20.3
UU2	Partially	62.1
UU3	Never	17.6
**VV**	**Copies of the Health and Safety Plan are sent to the subcontractors**	74.0
VV1	Yes	14.9
VV2	No	41.9
VV3	Sometimes	43.2
**WW**	**Conoce al Coordinador de Seguridad y Salud de la obra**	76.0
WW1	Yes	42.1
WW2	No	57.9
**XX**	**The on-site Health and Safety Coordinator is known ^1^**	38.0
XX1	Not known	7.9
XX2	Once a month	5.3
XX3	Once a fortnight	15.8
XX4	One or two days a week	21.1
**YY**	**The developer evaluated the health and safety measures that the contractor company would undertake when awarding the work.**	74.0
YY1	Yes	16.2
YY2	No	29.7
YY3	Sometimes	54.1
**AAA**	**Safety meetings were held with the subcontractors to disclose the progress and incidents of on-site safety conditions**	79.0
AAA1	Yes	15.2
AAA2	No	46.8
AAA3	Sometimes	38.0
**BBB**	**Who paid the Health and Safety Coordinator’s fees**	49.0
BBB1	Contractor	44.9
BBB2	Developer	32.7
BBB4	Contractor and developer	22.4
**CCC**	**Do you consider that the creation of a Classification of Contractor Companies, based on criteria of quality and management of workplace risk prevention would contribute favorably to improving safety conditions on the worksite?**	81.0
CCC1	Yes	51.8
CCC2	No	2.5
CCC3	Probably	45.7
**DDD**	**When you subcontract a worksite activity, do you evaluate the subcontractor based on their levels of safety?**	80.0
DDD1	Yes	10.0
DDD2	No	42.5
DDD3	Sometimes	47.5

^1^ The other categories were not included as many were not representative.

**Table A7:** Discrimination measures of the variables in each dimension.

Variables	Dimension		Mean
	1	2	
A	0.143	0.155	0.149
B	0.382	0.264	0.323
C	0.357	0.487	0.422
D	0.157	0.330	0.244
E	0.182	0.639	0.411
F	0.162	0.596	0.379
G	0.167	0.144	0.156
H	0.342	0.065	0.203
I	0.398	0.116	0.257
J	0.398	0.337	0.368
K	0.594	0.365	0.480
L	0.610	0.021	0.316
M	0.621	0.123	0.372
N	0.423	0.271	0.347
O	0.399	0.308	0.354
P	0.135	0.334	0.234
Q	0.098	0.138	0.118
R	0.333	0.099	0.216
S	0.113	0.121	0.117
T	0.365	0.332	0.349
U	0.665	0.162	0.413
V	0.717	0.329	0.523
W	0.574	0.195	0.384
X	0.658	0.183	0.420
Y	0.620	0.434	0.527
Z	0.673	0.382	0.528
ZZ	0.695	0.213	0.454
AA	0.492	0.375	0.434
BB	0.558	0.264	0.411
CC	0.523	0.247	0.385
DD	0.276	0.198	0.237
EE	0.480	0.157	0.318
FF	0.196	0.214	0.205
GG	0.568	0.369	0.469
HH	0.176	0.256	0.216
II	0.721	0.114	0.418
JJ	0.524	0.100	0.312
KK	0.728	0.143	0.436
LL	0.623	0.073	0.348
MM	0.232	0.216	0.224
NN	0.427	0.304	0.366
OO	0.005	0.043	0.024
PP	0.511	0.446	0.478
QQ	0.437	0.194	0.315
RR	0.187	0.146	0.166
EEE	0.043	0.018	0.030
SS	0.409	0.334	0.372
TT	0.130	0.579	0.355
UU	0.277	0.304	0.291
VV	0.339	0.305	0.322
WW	0.516	0.358	0.437
XX	0.115	0.364	0.239
YY	0.328	0.366	0.347
AAA	0.805	0.451	0.628
BBB	0.050	0.253	0.151
CCC	0.178	0.141	0.160
DDD	0.284	0.285	0.284
Active Total	22.122	14.765	18.443
